# Cultural diversity teaching and issues of uncertainty: the findings of a qualitative study

**DOI:** 10.1186/1472-6920-7-8

**Published:** 2007-04-26

**Authors:** Nisha Dogra, James Giordano, Nicholas France

**Affiliations:** 1 University of Leicester Greenwood Institute of Child Health, Leicester UK; 2 Center for Clinical Bioethics, and Division of Palliative Medicine, Georgetown University Medical Center, Washington, DC 20057, USA; 3 Samueli Institute, Alexandria, VA 22314, USA; 4 Neonatal Intensive Care, Guy's and St. Thomas' Paediatric Rotation, London, UK

## Abstract

**Background:**

There is considerable ambiguity in the subjective dimensions that comprise much of the relational dynamic of the clinical encounter. Comfort with this ambiguity, and recognition of the potential uncertainty of particular domains of medicine (e.g. – cultural factors of illness expression, value bias in diagnoses, etc) is an important facet of medical education. This paper begins by defining ambiguity and uncertainty as relevant to clinical practice. Studies have shown differing patterns of students' tolerance for ambiguity and uncertainty that appear to reflect extant attitudinal predispositions toward technology, objectivity, culture, value- and theory-ladeness, and the need for self-examination. This paper reports on those findings specifically related to the theme of uncertainty as relevant to teaching about cultural diversity. Its focus is to identify how and where the theme of certainty arose in the teaching and learning of cultural diversity, what were the attitudes toward this theme and topic, and how these attitudes and responses reflect and inform this area of medical pedagogy.

**Methods:**

A semi-structured interview was undertaken with 61 stakeholders (including policymakers, diversity teachers, students and users). The data were analysed and themes identified.

**Results:**

There were diverse views about what the term cultural diversity means and what should constitute the cultural diversity curriculum. There was a need to provide certainty in teaching cultural diversity with diversity teachers feeling under considerable pressure to provide information. Students discomfort with uncertainty was felt to drive cultural diversity teaching towards factual emphasis rather than reflection or taking a patient centred approach.

**Conclusion:**

Students and faculty may feel that cultural diversity teaching is more about how to avoid professional, medico-legal pitfalls, rather than improving the patient experience or the patient-physician relationship. There may be pressure to imbue cultural diversity issues with levels of objectivity and certainty representative of other aspects of the medical curriculum (e.g. – biochemistry). This may reflect a particular selection bias for students with a technocentric orientation. Inadvertently, medical education may enhance this bias through training effects, and accommodate disregard for subjectivity, over-reliance upon technology and thereby foster incorrect assumptions of objective certainty. We opine that it is important to teach students that technology cannot guarantee certainty, and that dealing with subjectivity, diversity, ambiguity and uncertainty is inseparable from the personal dimension of medicine as moral enterprise. Uncertainty is inherent in cultural diversity so this part of the curriculum provides an opportunity to address the issue as it relates to pateint care.

## Background

Ambiguity is defined as 1) a double meaning that is either deliberate or caused by inexactness in description, or as 2) an expression that is interpretable in more than one way [1]. Uncertainty is a fact or condition that lacks firm predictability, and can also refer to the condition of lacking certainty about a matter or circumstance [1]. While the two words have different meanings, in medical practice the use of either implies a lack of clarity and/or insecurity in constructs inherent to the clinical decisional process. Questions therefore arise as to what are the qualities and domains of ambiguity and uncertainty that are relevant and perhaps inherent to clinical practice, how are these manifest, and how should these be addressed in medical pedagogy and training.

There have been several studies investigating medical students' tolerance of ambiguity. These seem to reflect distinctions based upon interactions between personal characteristics, educational background and experience(s), and field of medical specialization.

DeForge and Sobey [2] administered Budner's [3] Tolerance for Ambiguity scale to entering 1^st ^year students in British medical schools for 4 consecutive years (n = 609) to investigate patterns of intolerance of ambiguity relative to demographic variables and initial preference of medical speciality. Medical students in this study were more intolerant of ambiguity than those first studied by Budner: students entering in 1985 were slightly more intolerant of ambiguity than those who entered in 1988. Students aged 23 or over were less tolerant of ambiguity than younger students. Male students were more intolerant than female, however both male and female students with natural/physical science undergraduate majors were more intolerant of ambiguity than their counterparts with humanities or social sciences backgrounds. Interestingly, medical speciality preference was not found to be related to intolerance of ambiguity. This suggests that students viewed medicine as a somewhat uniformly certain enterprise. As well, students who are educationally inculcated with the value-ladeness and apparent benefits of medical technology may become overtly reliant upon objective data, and develop a false sense of security about the certainty that such technically-gained objectivity conveys. These results illustrate that selection bias toward students with such applied scientific backgrounds may contribute to a pervasive technocentricism and technophilia, and with it cognitive dissonance toward appreciating the presence and value of subjectivity and/or uncertainty in medical practice.

Geller et al [4] studied 386 British medical students in years 1 through 4 using a modification of Budner's [3] Tolerance for Ambiguity scale. The study focused upon students' perceived tolerance for ambiguity in diagnosing and treating alcoholism (as exemplar of a clinically 'ambiguous' condition in that there are distinct perspectives that differentially regard/accept alcoholism as a "medical" versus social condition). It was found that tolerance for ambiguity: 1) did not change over time (i.e. – basic science students years one and two compared with clinical science students in years three and four); 2) was higher among students who wanted to become psychiatrists than those who desired careers in surgery; 3) was lower among men, whites and students who were younger when they began medical school, and 4) was lower among students who did not feel responsible for diagnosing and treating alcoholism. The last finding suggests that students who hold more distinct views about what is considered to be or not be a medical condition (e.g. in this case, alcoholism) are less able to tolerate ambiguity. This also suggests that certain students may find the subjective domains of patient interaction (e.g. narrative, individual psycho-social dimensions of illness experience, etc) more difficult to apprehend, interpret and/or incorporate into schema of clinical reasoning. This is supported by a study which found that fifth year British medical students who held positive attitudes towards psychiatry as a career choice were more tolerant of ambiguity (as measured by the complexity scale) than students with negative attitudinal regard of psychiatry [5]. Medical students interested in organic aspects of illness were less tolerant of ambiguity than students who were interested in a broader spectrum of both psychological and organic factors. This suggests that potential internists perceived general medicine as being a more certain field than psychiatry, and/or that potential psychiatrists were more comfortable with accepting the lack of certainty as inherent to their chosen field.

If medical schools admitted students possessing a high(er) tolerance for ambiguity (e.g. students with humanities and/or social sciences backgrounds), then the quality of care for particular conditions that are somewhat more ambiguous (e.g. chronic diseases, and/or psychiatric disorders and their respective illness dimension(s)) might improve, although it is debatable whether other fields of medicine actually manifest any greater level of certainty [6]. As McMullin, [7] and numerous others have noted (for review, [8]), the practice of medicine has always been characterized by uncertainty; a basic assumption being that uncertainty and ambiguity are related concepts that are constituent to the subjectivity that is at least part of medical assessment and diagnostic formulation [9,10]. Multiple types and domains of knowledge contribute to effective clinical reasoning, decision-making and the provision of care that is both biomedically correct and ethically sound [11,12]. In this way, the practice of medicine is a *tekne*, an art and skill that incorporates objective data and subjective apprehension of each particular patient in rendering good care [13]. Very often, it is the specific subjective context of objective information that creates ambiguity. But, this is what depicts the very predicament of illness as a first-person experience, laden with uncertainty that creates what Cassell [14] has termed the 'subjective portrait' of each patient that must be understood by the physician. As Reiser [15] has noted, much of medicine (*qua *humanitarian act) begins where technology ends, and this terrain of the clinical encounter is characterized by subjectivity and ambiguity. Medicine, by its nature is both inter-personal and fallible [16]. Thus, the physician must both understand (objective data and the fact that uncertainty can, and will occur), and be understanding (to the subjective nature of each patient, and their inherent, relative ambiguities) [17-19].

The changing demographics in both the UK and US have led to cultural diversity education gaining increasing attention [20,21]. Given the uncertainty that is inherent to cultural diversity, it is arguable that when teaching about cultural diversity, the issue of uncertainty in clinical practice cannot be avoided irrespective of student discomfort. Kai *et al *[22] engaged 9 focus groups consisting of 55 medical learners, including undergraduate students in a UK medical school and a group of postgraduate general practitioners in training to examine patterns of tolerance for ambiguity and uncertainty. The results indicated that there is a predominant 'difference' perspective, which might drive a narrow focus upon learning cultural knowledge, but that students seek to acquire and develop cultural knowledge as generic cultural 'skills', generally without self-reflection upon existing attitudes and dispositions. This supports the notion that students want to imbue their study of humanistic aspects of medical care with the (supposed) certainty that much of other medical training seeks and claims to provide. Thus, students (and often their teachers) expect to deal with diversity using the same level of certainty as they might in other, more technologically value-laden, scientific subjects (e.g. – clinical biochemistry, cellular pathology, radiology, etc).

This report is part of a PhD thesis [23] on the views held by key stakeholders in medical education relating to teaching and learning of cultural diversity. This paper reports on those findings specifically related to the theme of uncertainty as relevant to teaching about cultural diversity. The focus is to identify how and where the theme of certainty arose in the teaching and learning of cultural diversity, what were the attitudes toward this theme and topic, and how these attitudes and responses reflect and inform this area of medical pedagogy.

## Methods

A qualitative approach was employed because of its orientation toward exploring the responses and some of the reasoning behind them.

### Devising the interview schedule

Some formalized structure was necessary to address the specific research questions relating to the understanding of cultural diversity, its teaching and assessment. Therefore a semi-structured interview, with mostly open-ended questions was used [24]. The interview schedule was based upon the literature in sociology, medical education, education and intercultural studies [23], previous research [20,25], clinical, educational and personal experience; earlier interviews with members of the GMC Education Committee responsible for the first edition of Tomorrow's Doctors [26] and an internet search of all UK medical school websites.

#### Interview schedule

After brief introduction, each interview was conducted in three parts:

Part 1: Collection of basic demographic data (age and gender), as well as description of medical role(s) and experience.

Part 2: Inquiry using four open-ended questions to which respondents answered freely and unprompted, with the interviewer providing clarification as necessary. The four questions were:

1. How do you understand the term "cultural diversity"?

2. What do you think should be taught at undergraduate level about cultural diversity?

3. What main topics do you think that cultural diversity teaching should encompass at undergraduate level?

4. How do you think cultural diversity should be taught?

Each interview continued in open-ended style, but focused on specific aspects of teaching such as learning outcomes, delivery methods, assessment, and influence on clinical practice and student perspectives.

Part 3: Specifically addressed ways in which respondents used or understood key terms such as race, ethnicity and multiculturalism.

Each interview concluded by asking respondents about their experience and/or training in cultural diversity.

The interview was piloted with two policy-makers and one diversity teacher, after which minor modifications were made to the schedule. A flexible design was used when delivering the interviews, allowing modifications as part of the research process [24]. The interview schedule and transcript(s) are available upon request to ND.

### Sample characteristics and size

The sample of participants in this study was selected to represent the diversity and variety of stakeholders, within certain parameters generated from the literature and as consistent with the aims of the research. Those interviewed were not simply representative (or presumed to be representative) of their 'group' but in most cases were key informants within their group and often were significant to other stakeholders outside of the group. This sample was not representative of various groups: demographic attributes such as race, age, gender, socio-economic status were not applied to the sample selected. 'Professional status', job-title or 'role' was the characteristic feature used to identify the sample. The sample was not random in that key individuals were targeted. The use of a broad range of stakeholders with different priorities was important to assure that various perspectives were explored. There were no exclusion criteria employed.

There were two stages of sampling; the first assessed different groups of stakeholders; the second sampled different individuals from these groups. Within the main groups, there was noted overlap: for example, some policymakers were also medical educators, practising clinicians or worked in patient forums. The sampling strategy also ensured that interviews continued until saturation was achieved. Using sampling and 'snowballing' techniques, a total of 61 individuals were interviewed. The sample group included:

• **Communication teachers **(n = 6): teachers responsible for communication skills training;

• **Curriculum heads **(n = 7): heads of medical education and curriculum committee members who implement policy;

• **Diversity teachers **(n = 7): teachers are responsible for developing and delivering cultural diversity;

• **Policymakers **(n = 18): members of organisations that decide or influence policy on medical education (e.g. General Medical Council);

• **Researchers **(n = 2): included researchers in 'cultural diversity' and associated areas actively teaching on ethnicity;

• **Students **(n = 7): medical students;

• **Users **(n = 7): included patients, patient representatives and advocates.

Table 1 provides the basic demographic information of the participants. Formal association with a medical school was defined as being employed by the school (including clinical NHS staff appointed as honorary teachers and external examiners), or being a student (at a UK medical school). Individuals from 14 of the 26 established medical schools in the UK were involved (two schools have campuses at two sites; therefore, 12 curricula were effectively covered). Members from eleven policymaking organisations and six medical disciplines were interviewed. Other 'clinical' #perspectives included pharmacy, social work, community youth work and nursing. Non-clinical participants represented sociology, anthropology, accountancy, research, and patient advocacy fields. Policymakers were the most likely to not respond.

**Table 1:** Summary demographics of participants

**Number**	**Ethnicity**	**Gender (M= male F = female)**	**Mean Age Range in years**	**Medical school connection**	**Currently in Clinical Practice**
**Communication teachers **(n = 6)	5 White British 1 mixed race	3 M 3 F	46–50	6	4
**Curriculum heads **(n = 7)	7 White British	4 M 3 F	46–50	7	5
**Diversity teachers **(n = 14)	13 White British 1 Indian	5 M 9 F	46–50	14	6
**Policymakers **(n = 18)	13 White 3 Indian 1 Bangladeshi 1 Pakistani	17 M 1 F	51–55	9	13
**Researchers **(n = 2)	2 White British	2 M	41–45	2	0
**Students **(n = 7)	6 White British 1 Indian	3 M 4 F	Under 30	7	In training
**Users **(n = 7)	4 White British 1 Black British 1 Indian 1 Pakistani	4 M 3 F	41–45	0	0
**TOTAL **(n = 61)	50 British 6 Indian 2 Pakistani 1 Bangladeshi 1 Black 1 Mixed Race	39 M 22 Female	46–50	45	28

### Procedure

Interviews lasted approximately an hour and were conducted in person, whenever possible (alternatively by telephone). Initial contact was made through a formal introductory letter which outlined the purpose of, and invited participation in the study. The letter also explicated the confidentiality of the interviews, and that the local research NHS ethics committee had approved the project. If there was no response, the initial letter was followed-up by email or by a second letter until the target number of sample subjects was achieved. No one was contacted more than twice if they failed to respond. Most of those who agreed to take part did so by letter or email; copies of this correspondence were kept and maintained as a testimonial of written consent. Interviews were audiotaped and transcribed verbatim.

### Researcher characteristics and participation in the interview process

This research was undertaken by a female physician (psychiatrist) of Indian origin, aged forty, raised and educated in the UK, who works as a senior clinical academic at an East Midlands medical school. Having undertaken the development of a module in 'cultural diversity', the researcher had professional familiarity and experience with the topic. These factors have been shown to exert potential influence over the conduct of qualitative analyses [27].

### Analysis

The interviews were taped and transcribed verbatim. The data was thematically analysed [28] and followed a series of systematic steps outlined by Miles & Huberman [29] which were:

1. Read the complete scripts and identified key terms, words, themes and issues

2. Reviewed field notes and identified further themes

3. Collated responses to each question and from these identified types of response to issues that the question was based on

4. Ran systematic searches using key words and identified themes overarching different issues

There was also some frequency coding of responses to enable comparison between groups [30]. Themes were identified by the author and discussed with the thesis supervisor to ensure credibility of the analysis. Key themes were identified from the transcripts as a whole and from collations of responses to specific questions. Systematic searches using key words were performed to identify themes that overarched with different issues such as certainty. In evaluating each interview, particular issues and words relevant to searches to identify whether different individuals had used particular terms in other parts of the interview were noted. This helped identify similar themes in different parts of the interview. The issues or questions raised were reviewed and grouped into appropriate themes. The themes were not generated until after all the transcripts had been read at least once to ensure that a complete picture was obtained rather than just selected insights or responses that supported personal bias.

Mason [31] described three levels of analysis:

• Literal reading (cf denotative meaning): the content of the data and that which is literally said by the respondents in interviews.

• Interpretive reading (cf connotative meaning): this involves the analyst in constructing or documenting a version of what the analyst thinks the data mean or represent; that is, what can be inferred from the data?

• Reflexive reading: this refers to theoretical reflection and reflexivity and concerns not only the researcher's role in the process of generation and interpretation of the data, but also the way the data are read with respect to theoretical bases.

All three methods of reading were relevant for the analyses conducted in this research. The responses to different parts of the schedule were compared to examine whether or not response patterns were consistent with the use of language. The researcher interpreted the contents in the context in which responses were shared. Uncertainty was not a theme that was asked about specifically but was identified as relevant through the above analytical process.

Several computerised programmes exist to assist with qualitative data analysis. Robson [27] highlighted the advantages and disadvantages of specialist qualitative data analysis (QDA) computer programmes. However, given that computer programmes can only help with analysis (as they do not perform the analysis), can take some time to learn, and the researcher's inexperience with qualitative studies of this size, it was decided that manual analysis would be undertaken, recognising the limitations of the human as analyst [27]. The number of interviews was manageable for manual analysis and, additionally, there was a need to be able to consider how the interviews read as a whole (a need to bear the context of the statement with regard to the interview as a whole). Manual analysis also enables the researcher to become more familiar with the data and potentially enable more reasoned interpretations.

## Results

### Contents of cultural diversity teaching

Although mainly a qualitative study, some quantitative data is included to indicate the spread of responses [30]. The research did not specifically ask about uncertainty. However, issues of teaching about uncertainty arose over several different parts of the interview such as when discussing the contents of programmes, teaching methods and student perspectives.

Views about the meanings and interpretations of the concept of 'cultural diversity' were obtained from how respondents defined this term, what they thought should be taught, and how. The views of a fifth of respondents with at least a member of each of the sample groups were clearly aligned with the '*cultural sensibility*' model view of 'cultural diversity' [32]. Although expressed differently, the view(s) underlying these responses was that individuals are multidimensional, and that identity is based on more than skin colour, any other single characteristic. This definition introduces uncertainty because single characteristics such as race or ethnicity cannot be used to predict patients' lifestyles or preferences. Three respondents defined 'cultural diversity' in a way that was consistent with *'cultural expertise'*, but then went on to use the term in ways that were consistent with the *'cultural sensibility' *models. The curriculum head whose response aligned with *'cultural sensibility' *frequently used this term later in the interview in a more *'cultural expertise' *way, but then reverted to talking about individuals. An example of responses consistent with *'cultural sensibility' *is:

*"I suppose I would see it *[cultural diversity] *as actually being to do with ethnicity, along with religion, social class, gender, perhaps disability and sexual orientation, the ways in which people are different... I really think that the idea, that you can learn about what your average Muslim families are, is as stupid as suggesting that I must eat roast beef and two veg because I'm white and middle class" *(R8: Curriculum head)

The majority of the sample (42) defined diversity as being broader than ethnicity, but still saw it as a group-based identity which was socially, rather than individually defined. An example of such an approach is the following observation:

*"You talk about different religious groups and different communities within that. We came up with was more cultural competence – how to feel confident and be competent dealing with people from different backgrounds, different sociological groups and different cultures." *(R19: Diversity teacher)

This approach does not necessarily mean that individual diversity was recognised. For four respondents the model with which their responses aligned was unclear. Only three respondents fitted a '*cultural expertise*' model approach more neatly with ethnicity being the main determinant of culture.

*"There are lots of different cultures, so I am just looking at cultural diversity as simply being integration of all those cultures in a society. England as such is no longer simple Caucasian people with long heritage in England, it's no longer that" *(R1: Communications teacher)

A clinical policymaker identified that ethnicity was not always the only difference between doctors and patients, and gave the example of class, while a researcher gave an example of poverty.

A communications teacher was unsure about how to define 'cultural diversity',

*"I get slightly irritated when the term cultural diversity is translated into ethnicity and that gets reduced even further to black and Asian, predominantly. I feel that that's a very much-reduced notion of cultural diversity, but I think we also have problems about what we might include in cultural diversity" *(R2: Communication teacher)

This highlighted the problem that respondents were not always comfortable with the way terms were used, but also struggled to identify more meaningful terms. Views about the meaning of cultural diversity have not been explored as such, but much American literature addressing cultural competence has acknowledged the lack of clarity in this area [33]. The findings confirmed that there are different views about the meaning of cultural diversity. Faculty understanding of diversity is likely to influence the level of uncertainty acknowledged or addressed in teaching developed in this area,

Many respondents gave broad responses to the question "*What do you think should be taught at undergraduate level about cultural diversity?" *and also stated how they thought 'cultural diversity' could or should be taught. None of the respondents separated their responses into knowledge, skills and attitudes. The main themes included:

• Learning about groups of others (22; 2 communication teachers; 2 curricular heads; 4 diversity teachers; 8 policymakers; 5 students and a user)

• Unsure as to what should be taught under the rubric of cultural diversity (5; 1 policymaker; 2 students; 2 users)

• Sociological issues (3 all non-clinical)

• A set of responses which were unclear (e.g. thinking that students needed to be taught about cultural diversity, but that they could learn about it just by living in a multicultural society, being a minority, or through observation of everyday life) (6)

Learning about groups of others implies a level of certainty about what is taught.

Six respondents from across the sample were very clear in stating that students should learn about people as individuals and 2 of these were users who emphasized that patients need to be seen as "...people rather than bodies".

*"If medical students think they know everything about say what an Asian woman's experience is and then placed that on to a particular Asian woman who may not have had that experience. They may just think that they know a lot, and actually not be prepared to open their ears and listen. The diversity of any particular group members and their experience can be enormous...the individual differences will be greater than the actual differences that you can attribute to cultural origin." *(R58: User)

Of note is that this respondent maintained that students are misguided by claiming certainty in understanding of their patient's experiences because they have learned about groups of people and assigned them particular characteristics.

In response to the more specific question: "*What main topics do you think that cultural diversity teaching should encompass at undergraduate level?" *key responses included:

• Race and other aspects of diversity (35; 3 communication teachers; 5 curricular heads; 11 diversity teachers; 8 policymakers; 1 researcher; 4 students and 3 users)

• An awareness of the issue (16; 1 communication teacher; 4 curricula heads; 3 diversity teachers; 4 policymakers; 2 students and 2 users)

• Communication skills (11; 3 curricula heads; 1 diversity teacher; 2 policymakers; 1 researcher, 3 students; and 1 user)

• Self-reflection specifically (9; 3 diversity teachers; 4 policymakers; 1 researcher and 1 student)

• Learn to ask the right kinds of questions (3; 1 communication teacher and 2 curricula heads)

Five respondents were specific about what should not be taught. Three (a communication teacher, a diversity teacher and a curriculum head) felt diversity teaching was not just about giving information about specific groups. A student felt that it should not be a forum for religious education, and a policymaker felt that using a 'cookbook approach' (that is bringing with it a degree of certainty) would be unhelpful. Only the communication teacher mentioned race and ethnicity. Those that focused on process suggested centering upon issues rather than upon groups:

*"I think I would see it more as encouraging and questioning attitude rather than some kind of rote learning of facts... I think there is a danger in, there is a difficult balance between giving people enough information that they feel sort of confident, but without giving them so much information they feel they understand other people just by virtue of the fact they can check a list of facts about them. So I think it would be more the approach that says 'you're the expert in you so tell me about you"' *(R8: Curriculum head)

This representative response seeks to place value on the experiential shaping of perspectives and also seeks to empower the patient. Even if information is taught, the challenge of what constitutes the *right *information and how much is enough, remains in question. Four respondents felt that 'cultural diversity' needs to be prioritized in the curriculum, and that the curricular contents should build on students' interests, values and concerns, while 2 stated that this approach may also involve some unlearning of extant biases and dispositions. In some ways, this can be viewed as a slight contradiction, (i.e. the desire to build on what they bring, but an equal need to also help them unlearn certain negative stereotypes and biases that they have brought). In practice, however, the starting point may need to emphasize students' understanding of existing values and expectations. Unless this is clearly elucidated, other learning processes may simply build upon or be refractory to these biases, and in either case would be unlikely to succeed [34].

Only a diversity teacher suggested that students could read texts to obtain background knowledge. This issue is particularly relevant if transformative or self-reflective models of learning are applied. In these models, students are guided in learning rather than simply being taught what they might need. There needs to be clarity about what students are taught in a formal setting, and what they might be expected to achieve through self-learning. However, it is unclear whether effective communication can be achieved in the absence of self-reflection, as effective communication requires individual acknowledgement of personal influences upon any subjective interaction. As well, there is a need to consider learning styles and applications. Preferred or perceived necessary styles may be superficial and the need for certainty may relate more to passing academic examinations than the realistic future practice of medicine.

Concerns were expressed by the same communication teacher who thought that teaching should focus on facts about specific ethnic groups, about students needing to learn what were seen as potential gaffes, and about enabling some certainty in clinical practice:

*"...Patients beliefs are very important aren't they and we are always encouraged to look at patients' beliefs and understandings... Most *[of my patients] *are Caucasian people, so there is not a problem from that point of view but when you do come across it I personally find it difficult because you are not familiar with all their cultural beliefs and habits. That can be a disadvantage, particularly if you are working in that kind of environment. What needs to be taught? I've said their beliefs, a bit about their culture so that you can understand what is appropriate and what is not appropriate within certain cultures. I mean at one practice I went to it is inappropriate to offer a handshake. That's an insult apparently, but I don't know that as fact, that's what I was told. I think it may be important about how you, ways it's appropriate to examine and who to examine and who has to be there. I've no idea about that. Those are the sort of things that I'm thinking about, everyday contact with different cultures and knowing what's appropriate and what's not appropriate and what behaviour they think is reasonable behaviour" *(R1: Communication teacher)

This response raises the question of how many of our own, individual 'norms' tend to be suspended so as to accept other, more broadly construed, societal 'norms'? How might we best prepare students for the inevitability of collision or conflict between values, principles and even socially-defined norms? Should we perhaps be asking students to consider how they might manage such situations, as there is unlikely to be a single correct response? Also, what information about *'their' *cultures is important and who makes that decision. This response also highlights how assumptions are made about 1) heterogeneity in groups (especially majority groups) and 2) if the health care provider and patient share the same ethnicity; this is presumed to impart an understanding of each other's perspective.

The need to create expert students who never make mistakes is consistent with the *'cultural expertise' *model [32]. The respondents who viewed 'cultural diversity' consistent with the *'cultural sensibility' *model [32] still felt under pressure to give students factual information:

*"...I think certainly what we sort of talk to students about is the fact that prejudice is a normal almost phenomenon and everybody has prejudices and it's important to be reflective about those and to understand them, not to let them as enshrined in good medical practice, the GMC's good medical practice, not to let them get in the way, you are entitled to your attitude, but you mustn't manifest it in behaviour, it could disadvantage somebody. So I think a lot of what we should be teaching about diversity is to do with attitudes and reflective practice, and then there are factual things I suppose, or broad kind of factual issues. I mean, you may want to come on to this, but I will say it now, one of the things that we struggle with is the students are always asking us for detail. How do you relate to a Muslim? What do you do if somebody is wearing a funny hat or a sari? And we've tried to set back from that and say that is actually missing the point. Notwithstanding that, it is important for a student or a doctor working in an area with a particular ethnic minority groups, or working with particular minority groups, to understand something about the culture" *(R23: Diversity teacher)

Only a few (2 communication teachers; 1 curriculum head and 2 diversity teachers) of the respondents were happy not providing facts in diversity teaching. There was less emphasis on individuals challenging their own perspectives about physicians, and an increased tendency to view physicians as a homogenous group. It was also held that part of the focus may be on normalising prejudice (i.e. acknowledging that most individuals have prejudices and that this is not abnormal) and not preaching or proselytizing to students against this prejudicial "normality". This respondent was also aware of the difficulties in resisting pressure from students to provide clear-cut answers and focus on information about groups. Arising from this was the notion that it would be difficult to use student ideas to challenge and engage subsequent critical thinking on diversity, ambiguity and uncertainty.

Some ideas reflected a practicality rather than any clear philosophical premises:

*"I don't think we can really expect every student to know everything about say different cultures, religiously and socially. I think it's getting them aware that there are differences and as doctors they have got to respond to those and they have got to look at their own prejudices and communication skills within those groups" *(R25: Diversity teacher)

This implies that time and curricular latitude were the factors that limited what could be taught. There was also a perceived hierarchy of needs to acquire knowledge about certain groups (i.e. large minority groups warranted more curriculum space, suggesting that the need for knowledge about other, perceived lesser groups were less of a priority):

*"... Because there could be some basics and some main groups because there are large groups of certain ethnic groups and certain cultural groups" *(R35: Policymaker)

This too is consistent with *'cultural expertise'*, which focuses on local needs and which may inadvertently be taught in ways that communicate stereotypes. In summary, there was some superficial similarity in what was considered to be necessary knowledge of diversity, with just over one-third focusing on process, and a similar number focusing upon groups. Only one-fifth of respondents focused on specific skills of self-reflection and/or modes of questioning. However, upon further assessment it was found that individual respondents communicated greater variety of foci, which may reflect individual concerns and/or priorities.

For the question, "*How do you think cultural diversity should be taught" *more than one response was possible. The following responses were made:

• Small group work to help students explore and discuss the issues (30; 4 communication teachers; 4 curricula heads; 11 diversity teachers; 7 policymakers; 3 students and 1 user)

• Lectures suggesting they wanted some information or facts to be taught (19; 1 communication teacher; 7 diversity teachers; 9 policymakers; 1 researcher and 1 student)

• Community placement (20; 2 communication teachers; 3 curricula heads; 2 diversity teachers; 6 policymakers; 4 students and 3 users) suggested community placements).

• Experience of actually talking to diverse communities (17; 2 communication teachers; 2 curricula heads; 5 diversity teachers; 4 policymakers; 3 students and 1 user)

• Clinical contexts (18; 3 communication teachers; 2 curricula heads; 4 diversity teachers; 3 policymakers; 2 researchers, 2 students and 2 users). Of these 9 were clinicians.

Some felt that lectures and other didactic teaching should support small group work but some (a diversity teacher, a policymaker and a student) felt that lectures had no place in 'cultural diversity' teaching. The justification for community placement was variable ranging from talking to individuals representatives of their community (1 policymaker and 1 student), to talking to those that are different from oneself in order to give students experience of the wider community (2 users). A student felt that experience can be good or bad and that experience in itself was not enough. He particularly wanted time for discussion and reflection.

*"I think they have actually got to DO it. They have got to practice doing difficult consultations. They have got to get feedback on their practice. They need to be supported and encouraged to feel confident and OK about themselves in order to be able to operate better with anybody" *(R2: Communication teacher)

This is consistent with students wanting an experiential approach. Students, however, did not mention theoretical underpinnings or other teaching to perhaps support the experiential learning. Two communication teachers, 2 diversity teachers and 1 student mentioned role-play which could be a safer method of offering experience. A communication teacher and a diversity teacher, both of whom had much experience of working with simulated patients in communication skills, touched on the expense of using simulated patients. One of these discussed the skills needed by simulated patients, and why they might be more appropriate than real patients in detail:

*"As a trained actor you get an increased awareness of yourself, so you work out what is you, the way you react and the way you impact on others, because through understanding that you can then learn how to take on somebody else and leave the bits of yourself behind whereas if you take a lay person who has never done acting training usually they will just stick on a hat and, it's not quite the same as believing... Actors allow them [students] to struggle enough so that the teaching points are there, but not so they are completely destroyed" *(R19: Diversity teacher)

This suggests that those participating know how to be effective facilitators as this may influence 'cultural diversity' teaching. Effective facilitators are more likely to engage students in constructive debate and encourage them to self-reflect and share their perspective than tutors who are didactic. There is also the opportunity to allow students to learn to deal with uncertainty.

### Inconsistencies in the contents of 'cultural diversity' programmes

There were noted inconsistencies between the concept and expectations of the educational process, learning outcomes and the final contents of programmes. Students' and some teachers' responses made clear that students often want or need information on diversity, that they can easily utilize to promote a sense of professional security (i.e. – certainty) when dealing with issues in cultural contexts. Few respondents stressed the importance of explicitly learning about ambiguities and uncertainties in clinical practice. Even when respondents indicated a need or desire to employ small group teaching to promote discussion about self- and general cultural attitudes, there was a sense that the use of an external 'expert' enabled such discussion. This 'expert' related the issue at hand, whether it be ethnically-, gender-, or sexual practice-based. The use of such extrinsic experts has the potential to externalise diversity and not necessarily challenge existing assumptions of teachers or students. It is arguable that the idea of "experts" in diversity meets political rather than educational aims. In this sense, it is of interest that no one raised the question of whether teaching about 'cultural diversity' is synonymous with teaching about equality.

Teaching about 'cultural diversity' was largely seen as playing a part in improving health care through improved communications, and not necessarily through better understanding of individual patient needs. Few teachers felt comfortable letting students struggle, although there were exceptions.

*"So, it doesn't worry me too much when the students struggle... I think we tread a tightrope between that sort of experiential learning, which in a way is my background, and the students' desperate need for certainty... Here's a person who is clearly from a different religious background and ethnic background, clothing what does that symbolise, what toes am I going to tread on? And there is a danger if they sort of learn half a recipe, it's worse, learn nothing, then just find out" *(R24: Diversity teacher)

*"Uncertainty, paradoxical things, chaos is very close to us and existing in that zone of uncertainty is actually what we are asked to do as doctors..." *(R45: Policymaker)

Students emphatically affirmed their belief that teaching about different religions and religious values was important. It should be noted, however, that this is easily accessible from written texts. Yet, while basic religious constructs and principles can be read, it is still preferable to directly relate to patients' spiritual needs, rather than make assumptions in this regard [35,36]. But how should this be accomplished? Students' desire for facts and certainty is consistent with what is provided elsewhere in the medical curriculum, and in reality it may be difficult for faculty (and administrators) to resist student pressure because to some extent, successes of educational outputs are judged through student feedback To reiterate, medical curricular development should be based upon defined needs' assessments, rather than simply focusing on the needs or wishes of the most vocal interest group(s). Given the relatively low status of 'cultural diversity' teachers and the lack of credibility of the subject within most medical programs [37], faculty may feel that by not acquiescing to the student demand for providing certainty, their positions are further undermined.

As well, leaders of particular socio-cultural groups may feel that a principle-based approach does not sufficiently address their concerns, thus, faculty may feel pressured to include information about several groups to demonstrate cooperativity and/or collaboration. Such political agendas may influence the educational programme more than educational ideas – explicating that "...all patients are individuals" but then reinforcing the importance of people belonging to groups might convey contradiction and undermine credibility. Such political influence (e.g. – interest or 'pressure' groups) may also force faculty and administrators to include contents that are inconsistent with schools' academic philosophy and/or demonstrated educational needs, thus leading to pedagogically inadequate, incoherent programmes.

### Student influences on the development of 'cultural diversity' teaching

Student perspectives were recognised as important, but there was concern about students' desire for certainty in those educational and practical domains where certainty is likely to be uncommon. Most assuredly, medical students need to understand the realities of medical practice and recognise that ambiguities are inherent to practice. Yet, the interviews suggested that a realistic depiction of uncertainty and ambiguity in practice was not being provided at earlier stages in medical education, despite the development of different types of curricula:

*"Dealing with uncertainty in medicine is a big issue. We do discuss some of this in our session, about dealing with uncertainty and sort of trying to get students to understand that actually most of their professional life they are going to be dealing with uncertainty, sadly.. But dealing with uncertainty is one of the things I am really interested in and of course cultural diversity just increases that uncertainty. I don't think it, in a sense for me, when, we are now thinking of teaching, not that we do a lot of teaching, hopefully the students do a lot of learning. I don't think we necessarily do a lot of teaching. It's very much a sort of facilitated programme that we operate from, but dealing with differences, whether they are cultural differences, however you define, because of course we haven't decided, on a consensus about what this means anyway" *(R2: Communication teacher)

## Discussion

The issue of uncertainty is highly relevant for teaching cultural diversity. This was evidenced by how often the theme of uncertainty arose in conversations that were focused upon cultural diversity education. To date much of diversity education has worked towards a model of 'cultural expertise', [23,38]. If cultural diversity training is to be patient-centred, then a different approach – one that acknowledges the uncertainty in clinical practice – may need to be developed [32,39]. Students may demonstrate an awareness of the issues raised but clearly have not uniformly demonstrated a willingness to be personally challenged. This was previously illustrated by Culhane-Pera et al [40] who have shown that physicians in training wish to receive concrete information from which they can generate 'do and don't' lists for use in clinical practice, and thus reiterated the need for medical faculty to resist simply providing students with such lists. This supports the notion that while students recognise that cultural diversity training is needed, and that such training and its effects can and often do occur beyond the classroom, they still expressed a strong desire for certainty-based, factual information [25,41]. We agree with Simpson et al. [42] that simple provision of such factual information is insufficient, given that clinical reasoning involves an element of uncertainty [43,44].

The Association of American Medical Colleges produced the Tool for Assessing Cultural Competence Training (TACCT) in order to provide guidelines and support for medical schools trying to implement cultural competence training [45]. The TACCT has five domains that emphasize knowledge, skill and attitudinal learning outcomes. The issue of uncertainty is not explicitly addressed in the TACCT. However, objectives such as exhibit comfort when discussing cultural issues implies helping students to learn to be comfortable with the uncertain or the unknown. Tervalon and Murray-Garcia [46] argued for a cultural humility approach which empowered the patient to relate their perspective, rather than have the physician make particular assumptions through learned expertise. This approach requires the physician to be comfortable with the uncertainty and ambiguity. If medical curricula are to become fully integrated (i.e. balance humanitarian and scientific values), diversity teaching might be more successful as students either 1) develop professional skills and become more aware of the notion of uncertainty in clinical practice, or 2) are recruited into medical programs based upon a selection bias that favours their acceptance of ambiguity and uncertainty (i.e. – those with humanities and social sciences' versus strictly natural/physical sciences undergraduate education). Weiss [47] argued that medical students are overwhelmed by new information and rarely appreciate patients' complaints beyond the inherent biomedical aspects. Emphasizing science, without acknowledging the importance of the humanities, undermines the essential attribute of the good physician – the ability to establish a productive dialogue with the patient [48]. Education in the humanities can broaden students' scientific perspective and reinforce the critical, interpretive and interpersonal tasks of medical hermeneutics. The humanities inculcate a tolerance for ambiguity, provide a basis for the reconciliation of competing values, and foster the ability to discern the narrative thread in the setting of illness. However, this is part of the wider debate that challenges the certainty and status with which the biological sciences are viewed compared with the psychosocial sciences within medicine and medical education [37].

Even after diagnostic and therapeutic regimens have been thoroughly studied, there will always be the fundamental uncertainty of medical practice: the fact that epidemiological research at best produces probabilistic results that cannot predict with complete accuracy the effectiveness of a given treatment for a specific patient. Park [49] argued that the providing complementary and alternative medicine topics in medical school curricula helps to elucidate the complex and uncertain nature of medical practice and increases cultural sensitivity, amongst other things. However, it is also arguable that a different approach may be to directly address the role and necessity of uncertainty in medicine. Given the constraints of curricular time, an appropriate way to address issues of uncertainty may be through cultural competency education that could be directly expanded as an existing facet of the medical curricula. Epstein and Hundert [50] maintain that management of ambiguity should be part of assessing for professional competence. There is an argument that a raised tolerance for ambiguity and uncertainty are important characteristics of effective communication – an individual tolerance for ambiguity affects the type of information we try to seek about others [51]. People with a low tolerance for ambiguity tend to gather information that supports their own beliefs. In contrast, those with a high tolerance for ambiguity may seek more sensitive, less biased information about others, in attempt to gain inter-subjective understanding as part of a broadly objective interpretation. This approach requires flexibility and an ability to tolerate uncertainty, which can create discomfort in some. If these issues are not addressed, students may accept the factual components of courses (and medicine on the whole), but fail to recognize the importance of other, less concrete, more esoteric aspects of information. Translating didactics into experience, this could lead to an over-reliance upon objective data, and an under-appreciation of subjective, contextual dimensions of patient interaction. In this case, 'cultural competence' teaching would stand to do nothing more than reinforce stereotypes, as health professionals try to resolve ambiguity by interpreting patient semiotics in ways which suit extant biases and predispositions, rather than being sensitive to what is being said and/or meant.

If curricula continue to teach specific factual information they not only miss a key opportunity to discuss certainty but may also collude with students that cultural diversity is about facts rather than self reflection, personal development and professionalism as suggested by several authors in the field [39,52].

It is also worth noting that if we are to teach students about uncertainty and cultural competence, then we need to consider how faculty and clinical staff are trained and mentored in this area.

## Conclusion

Students may feel that cultural diversity teaching is more about how to avoid professional, medico-legal pitfalls, rather than improving the patient experience or the patient-physician relationship. Inadvertently, medical education may foster and accommodate this misinterpretation, both through selection and training biases. While technology can, and frequently should be used in diagnosis and treatment, we maintain that it cannot establish the subjectivity and humanitarian appreciation that is the conduit for the medical relationship. Uncertainty predicates each dialogical relationship and the clinical encounter is no different, for each patient is unique, and therefore diversity teaching is as much about understanding individuals as it is about understanding groups and cultures. Thus, while we must teach medical students to use the most advanced technology in application of the science of biomedicine, we opine that it is of equal and perhaps greater importance that we teach our students that technology cannot guarantee certainty, and that dealing with uncertainty and ambiguity is inseparable from the personal dimension of medicine as a moral enterprise.

## Competing interests

The author(s) declare that they have no competing interests.

## Authors' contributions

ND conducted the study and analysed the data. JG and NF contributed to the writing of the paper. All the authors read and approved the final manuscript

## Pre-publication history

The pre-publication history for this paper can be accessed here:


